# Could Capsule Endoscopy Be Useful in Detection of Suspected Small Bowel Bleeding and IBD-10 Years of Single Center Experience

**DOI:** 10.3390/diagnostics14090862

**Published:** 2024-04-23

**Authors:** Jelena Martinov Nestorov, Aleksandra Sokic-Milutinovic, Aleksandra Pavlovic Markovic, Miodrag Krstic

**Affiliations:** 1 School of Medicine, University of Belgrade, 11000 Belgrade, Serbia; asokicmilutinovic@gmail.com (A.S.-M.); akica68@yahoo.com (A.P.M.); krstic.miodrag61@gmail.com (M.K.); 2 Clinic for Gastroenterology and Hepatology, University Clinical Center of Serbia, 11000 Belgrade, Serbia

**Keywords:** video capsule endoscopy, small bowel, obscure gastrointestinal bleeding

## Abstract

A retrospective study in patients who underwent video capsule endoscopy (VCE) between 2006 and 2016 was conducted in the Clinic for gastroenterology and Hepatology, University Clinical Center of Serbia. A total of 245 patients underwent VCE. In 198 patients the indication was obscure gastrointestinal bleeding (OGIB), with 92 patients having overt and the other 106 occult bleeding. The remaining 47 patients underwent VCE due to suspected small bowel (SB) disease (i.e., Von Hippel–Lindau syndrome, familial adenomatous polyposis, Peutz Jeghers syndrome, Crohn’s disease, prolonged diarrhea, abdominal pain, congenital lymphangiectasia, protein-losing enteropathy, tumors, refractory celiac disease, etc.). VCE identified a source of bleeding in 38.9% of patients (in the obscure overt group in 48.9% of patients, and in the obscure occult group in 30.2% of patients). The most common findings were angiodysplasias, tumors, Meckel’s diverticulum and Crohn’s disease. In the smaller group of patients with an indication other than OGIB, 38.3% of patients had positive VCE findings. The most common indication is OGIB, and the best candidates are patients with overt bleeding; patients with IBD should be evaluated in this setting.

## 1. Introduction

Video capsule endoscopy (VCE) is a diagnostic method that enables the non-invasive, direct visualization of the entire small bowel (SB). A complete exploration of the small bowel is challenging and difficult due to its length and tortuosity [1]. Standard endoscopic procedures (esophagogastroduodenoscopy, ileocolonoscopy) provide access to a small segment of the proximal and distal parts of the small bowel. Explorations using push enteroscopy or intraoperative enteroscopy are both invasive procedures that do not always allow for the visualization of lesions in the small bowel. Small bowel barium follow-through (SBFT) radiography has low sensitivity and nowadays has been mostly replaced by newer, more advanced radiological methods such as CT and MR enteroclysis/enterography. CTs and/or MR enterographies are used to assess the transmural inflammation of the intestine and have limited sensitivity for superficial lesions [2,3,4]. Together with the discovery of VCE came the development of “double balloon” enteroscopy (DBE). VCE and DBE led to a revolutionary breakthrough and significant progress in examining the SB. VCE is a simple, safe, noninvasive procedure, well-tolerated by the patient, and it is referred to as “physiological endoscopy” since the capsule moves passively, does not inflate the bowel, and records images of the mucosa in the collapsed state [5,6]. The initial VCE developed by Given Imaging (Yoqneam, Israel) was approved in Europe by the European Medicines Agency (EMA) and in the United States by the Food and Drug Administration Agency (FDA) in 2001. Since then, almost 20 years have passed, and VCE has evolved rapidly due to important technical improvements and the increased availability of equipment from different manufacturers [6].

In this article, we report 10 years of experience with VCE in the Clinic for Gastroenterology and Hepatology, University Clinical Center of Serbia, which served as the single referral center in Serbia until 2008. The main indication for the use of VCE has been obscure gastrointestinal bleeding (OGIB), as well as suspected Crohn’s disease.

## 2. Materials and Methods

A total of 245 VCE examinations were performed from October 2006 until March 2016 in the Clinic for Gastroenterology and Hepatology, University Clinical Center of Serbia. The main indication for VCE was obscure gastrointestinal bleeding (OBIG), while a number of patients were examined with other indications such as unexplained chronic abdominal pain, chronic diarrhea, suspected Crohn’s disease, suspected small bowel tumors, protein-losing enteropathy, Von Hippel–Lindau syndrome, familial adenomatous polyposis (FAP), Peutz–Jeghers syndrome, congenital lymphangiectasia, eosinophilic gastroenteritis, etc. OGIB is defined as a bleeding of unknown origin that persists or recurs after an initial negative upper and lower gastrointestinal endoscopy. Patients with OGIB were further classified into two categories:

(1) overt obscure bleeding if they had clinical signs of gastrointestinal bleeding (melena, hematochezia).
(2) occult obscure bleeding if there were no clinical signs of gastrointestinal bleeding in the presence of iron deficiency anemia with a positive fecal occult blood test [7].

The GIVEN Video Capsule system (Given Imaging, Yoqneam, Israel) was used with M2A/SB capsules. The real-time viewer (Given Imaging) was used from 2010 until 2016. The day before the VCE, each patient received bowel preparation with oral purge −2 L polyethyleneglycol-based solution. Intravenous metoclopramide (10 mg) was also given to all patients 30 min before swallowing the capsule. Patients were allowed to drink clear liquids 2 h and eat light food 4 h after swallowing the capsule. In pediatric patients, a capsule was delivered into the duodenum using an endoscope. The recorder of the VCE was disconnected only after the battery stopped blinking, approximately 8–11 h after capsule ingestion. Only one procedure had technical difficulties, with the capsule not becoming active after removal from the container and being replaced with another capsule. All other patients had a smooth examination.

Two gastroenterologists carried out the interpretation of images. Lesions were classified according to the Saurin score into three categories. Those having no potential for bleeding were classified as P0 lesions, those having uncertain hemorrhagic potential (such as red spots on the intestinal mucosa or small isolated erosions) were classified as P1 lesions, and those having a high potential for bleeding (such as typical angiomata, large ulcerations, tumors, or varices) were classified as P2 lesions [8].

Patients were asked to note the evacuation of the capsule, and those who were suspected of having retained the capsule (suggested by the capsule interpretation) or who were uncertain or concerned were given serial X-ray/fluoroscopic screenings at weekly intervals. Depending on the VCE finding, some patients were also followed up in the form of medical therapy (such as treatment for Crohn’s disease, a gluten-free diet, or the institution of antihelminthic therapy), surgical therapy (for tumors or Meckel’s diverticulum), or enteroscopy evaluation using a Fujinon EN-450T5 double balloon enteroscope (ulcers, polyps, or bleeding angiodysplasia). Those with negative VCE reports were followed up for one year after the VCE examination. The study was conducted in accordance with the Helsinki declaration.

In the statistical analysis, categorical data were presented as absolute and relative numbers in percentages. Numerical data were described with mean and range from minimum to maximum. For testing the difference in the distribution of nominal data categories, the chi-square test was applied. Statistical methods were considered significant for the level of 0.05. The complete analysis was performed in the statistical software IBM SPSS version 21.

## 3. Results

OGIB was the most common (198/245 patients; 80.8%) indication for VCE during the study period. Out of 198 patients with OGIB (109 males, 89 females, mean age 51 ± 20 (median 53; age range 3–83 years), 106 patients had obscure occult bleeding and 92 obscure overt bleeding. The median duration of symptoms was 10 months in patients with OGIB (range 1–108 months). Hemoglobin was 98.47 ± 18.51 g/L, ranging from 60 to 151 g/L (Table 1).

The overall diagnostic yield of VCE in our study was 66.2% and was calculated using all P2 and P1 lesions detected. VCE identified a source of bleeding (P2 lesions) in 77/198 (38.9%) with OGIB (occult and overt) and other SB pathologies as seen in Table 2. In the subgroups of OGIB, VCE identified a source of bleeding (P2 lesions) in 45/92 (48.9%) patients with overt bleeding and in 32/106 (30.2%) patients with occult bleeding. P1 lesions were seen in 22/92 (23.9%) patients with overt bleeding and in 32/106 (30.2%) patients with occult GI bleeding. In 25/92 (27.2%) patients with overt bleeding and in 42/106 (39.6%) patients with occult bleeding, VCE lesions were classified as P0. There was a statistically significant difference in the frequency of P2, P1, and P0 lesions between OGIB patients with occult and with overt bleeding (*p* = 0.025). There was a significantly larger number/percentage of P2 lesions among OGIB patients with overt bleeding, while P0 lesions were more common among OGIB patients with occult bleeding. There was a statistically significant difference in the frequency of P2 lesions between OGIB patients with occult and overt bleeding (*p* = 0.007). There were significantly more P2 lesions among OGIB patients with overt bleeding than among OGIB patients with occult bleeding.

**Figure 1:**
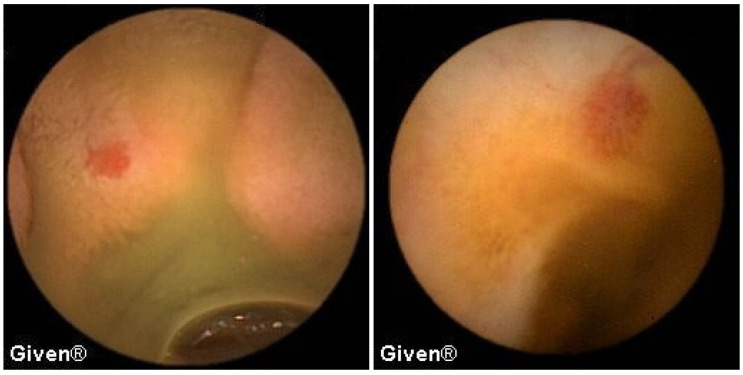
Jejunal angiodysplasias.

**Figure 2:**
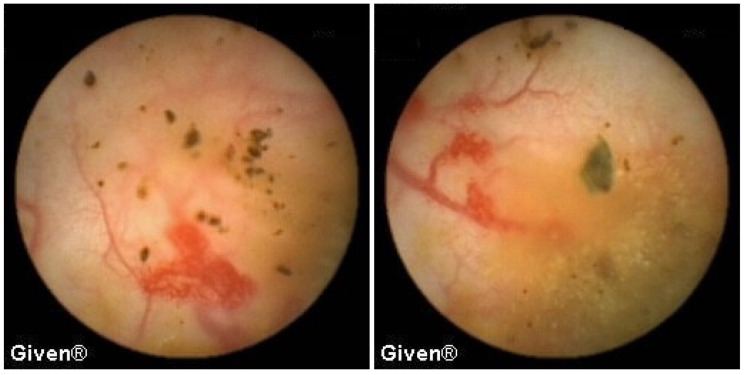
Cecal angiodysplasias previously missed in colonoscopy and diagnosed using VCE.

A detailed analysis of underlying SB pathology is displayed in Table 3. In patients with OGIB, the most common P2 lesions in both subgroups were vascular lesions, mainly angiodysplasias localized in the small intestine (mostly in the duodenum and proximal/medial jejunum), but in two patients angiodysplasias were observed in the cecum (previous ileocolonoscopies were negative). After the capsule examination, 24 patients with angiodysplasias underwent argon plasma coagulation (APC) of lesions accessible to the DBE. After intervention, none of them had further episodes of bleeding (Figure 1 and Figure 2). The most common P1 lesions were isolated vascular lesions.

**Figure 3:**
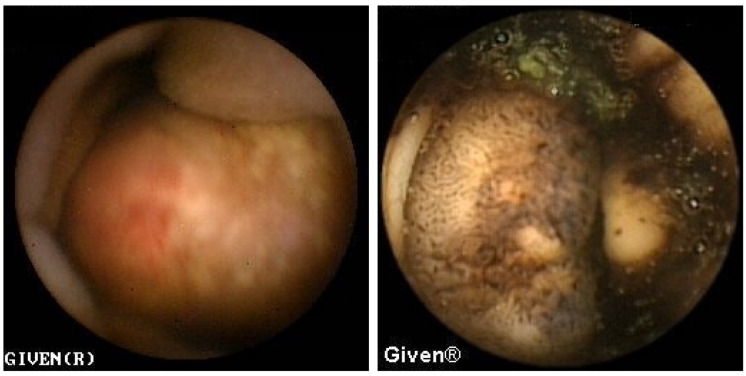
Gastrointestinal stromal tumor (GIST) in the small bowel.

**Figure 4:**
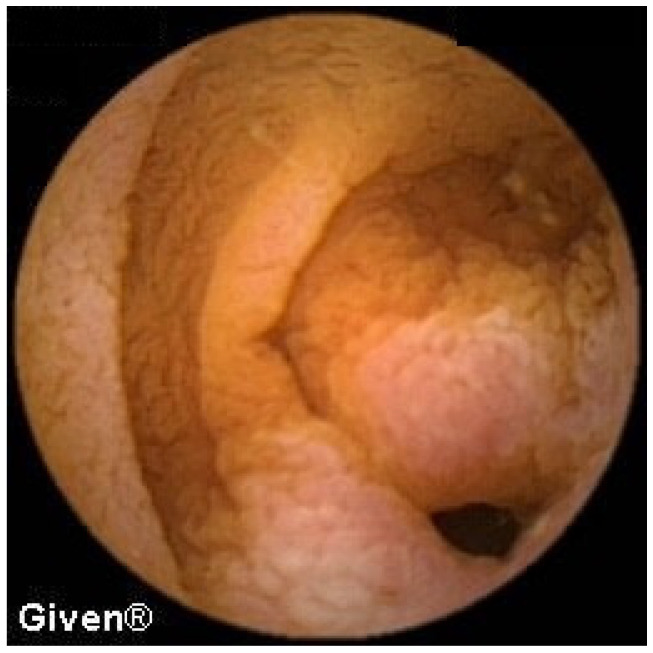
NHL of the jejunum.

**Figure 5:**
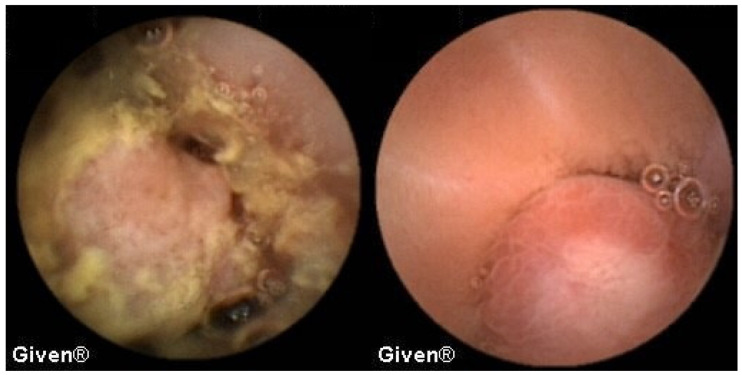
Metastases of malignant melanoma.

**Figure 6:**
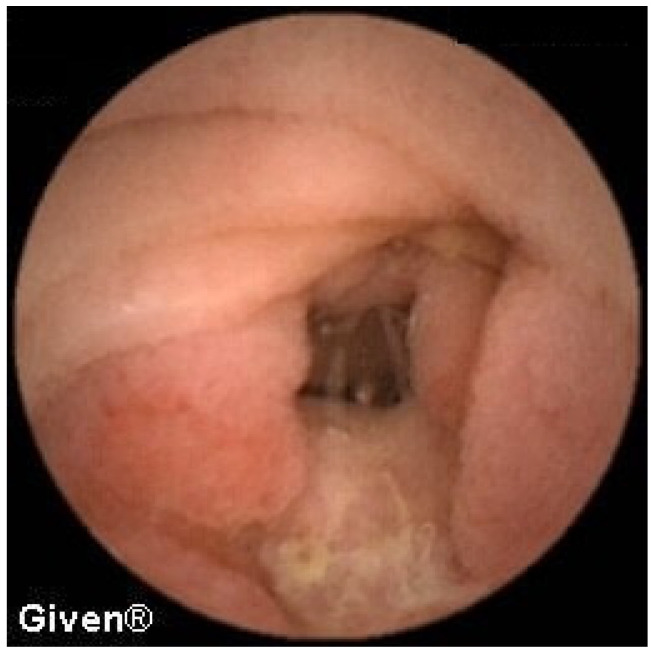
Adenocarcinoma of the proximal jejunum.

**Figure 7:**
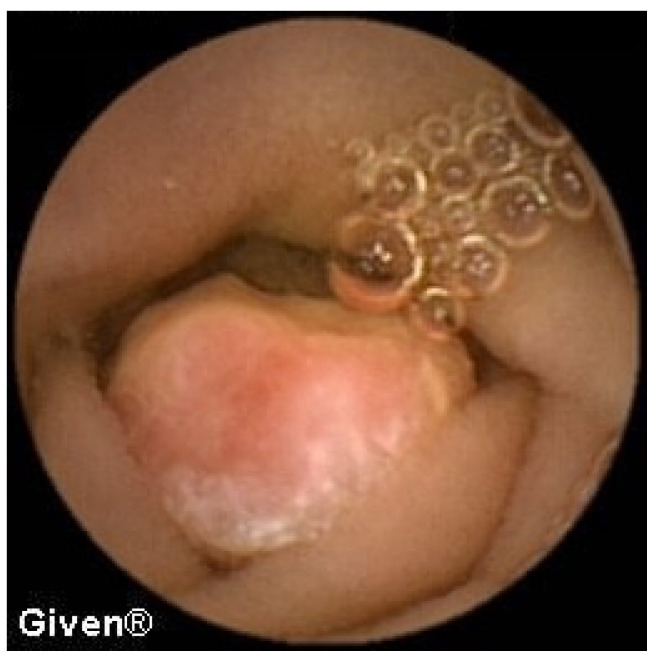
Leomyosarcoma of the medial ileum.

Out of 22 patients in whom the SB tumor was diagnosed, seven patients were operated on with the histological confirmation of gastrointestinal stromal tumor (GIST), as seen in Figure 3. In three patients, a neuroendocrine tumor was histologically confirmed, while two patients were diagnosed with Non-Hodgkin’s lymphoma (NHL) (Figure 4). Metastases of malignant melanoma were verified histologically in three patients (Figure 5), and in two patients histology confirmed adenocarcinoma of the proximal jejunum (Figure 6). In a single patient, VCE revealed a tumor of the medial ileum later histologically classified as leiomyosarcoma (Figure 7). Four patients, in whom tumors were diagnosed, did not accept suggested surgical exploration or further follow-up.

**Figure 8:**
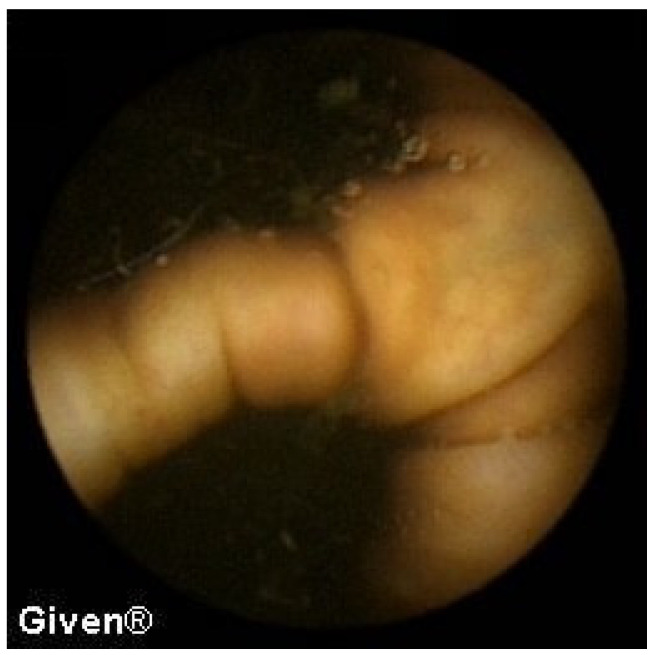
Double lumen sign in a patient with Meckel’s diverticulum.

In 13 patients, at the level of the medial/distal ileum, we noted clear signs of the presence of two lumens, which in terms of localization and morphology corresponded best to the existence of Meckel’s diverticulum. The most common signs of MD on VCE include, as we previously reported [9], double lumen signs, visible blood, and diaphragm signs (Figure 8). The positive predictive value for diagnosing MD using VCE in our case series was 84.6%, as per our previous report [9]. In all thirteen patients the diagnosis of MD was confirmed during subsequent surgery.

**Figure 9:**
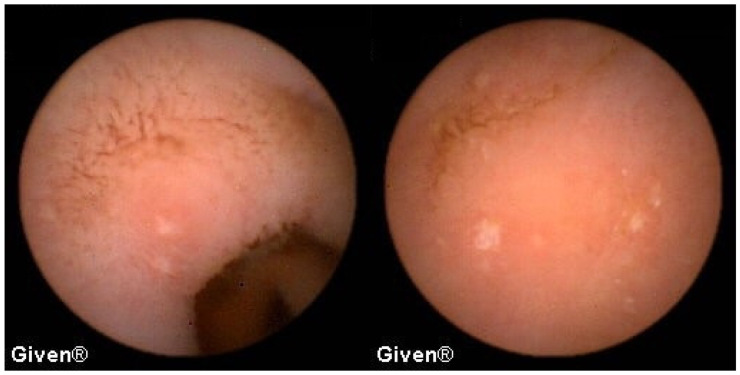
Subtle aphtous lesions that can be missed with other diagnostic tools.

**Figure 10:**
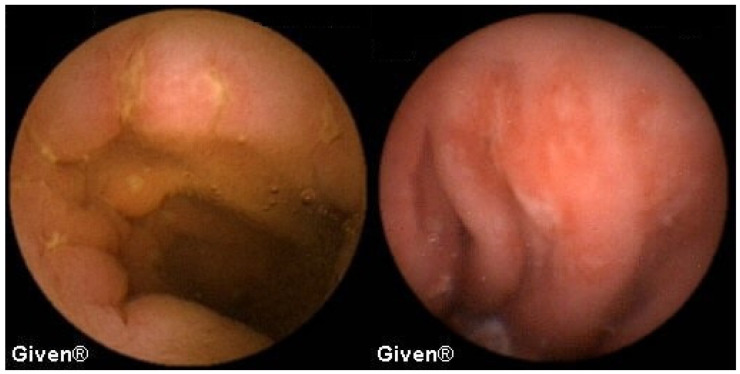
Serpiginous and linear ulcerations in the small bowel.

In three patients, multiple aphthous and serpiginous ulcerations, typical of Crohn’s disease, were seen in the jejunum and ileum and subsequently confirmed histologically during DBE as newly diagnosed Crohn’s disease. The patient denied previous NSAID use, which is clinically relevant information because NSAIDs can induce similar mucosal lesions in the SB. An appropriate therapy was prescribed to all patients, and clinical remission was achieved (Figure 9 and Figure 10). Meckel’s diverticulum was diagnosed in one patient with Crohn’s disease.

A finding similar to the one seen in Figure 9 with multiple erosions in the jejunum and ileum, without signs of bleeding, was detected in three patients with a history of frequent NSAID use.

**Figure 11:**
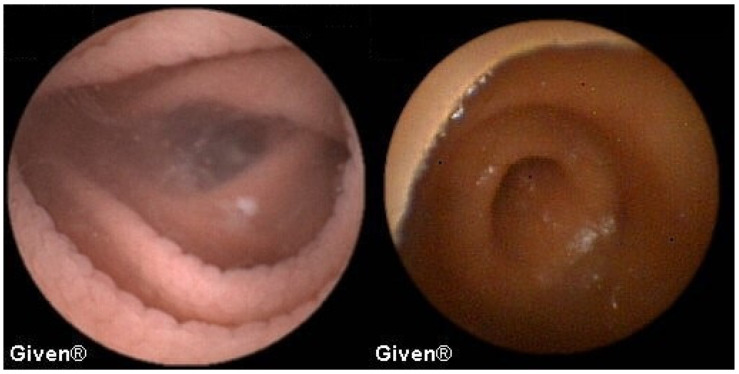
Celiac disease in the proximal jejunum (**left**) and ‘patchy’ celiac disease in the ileum (**right**).

Mucosal atrophy and a mosaic-like mucosa—a typical finding for gluten-sensitive enteropathy—was observed in the proximal jejunum in two patients, while in one patient part of the ileum was also affected (Figure 11). In these patients, previous endoscopic and histological findings of duodenum and proximal jejunum were completely normal. The patients were put on a gluten-free diet, and they responded with complete clinical recovery and normalization in their hemoglobin level.

**Figure 12:**
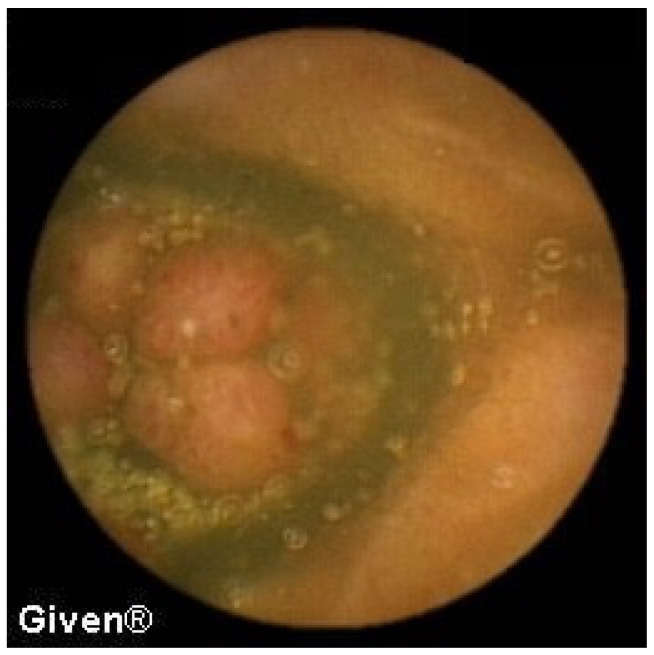
Polyp in the terminal ileum.

In one patient, a large polyp occupying ¾ of the intestinal lumen with multiple erosions was observed in the terminal ileum (Figure 12). The patient did not accept further diagnostic algorithm, the proposed surgical intervention, or further follow-up.

**Figure 13:**
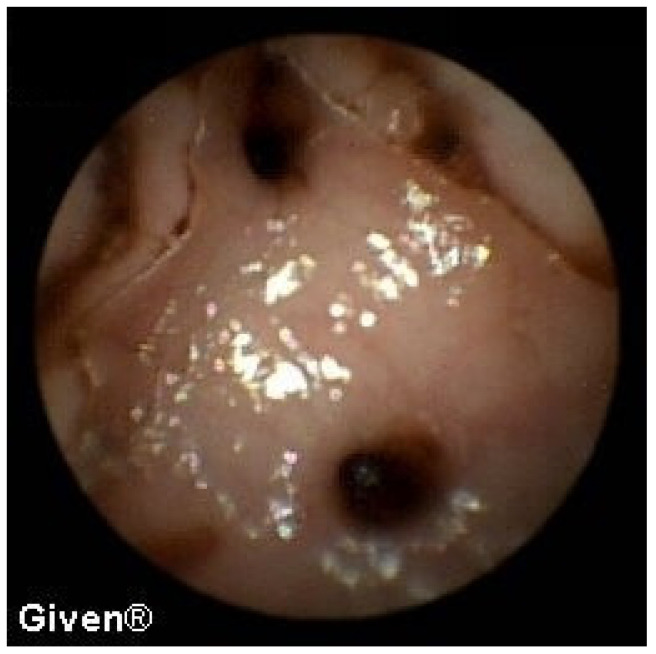
Cecal bleeding diverticulosis.

Active bleeding from the cecal diverticulum, which was not diagnosed during the previous ilecolonoscopy, was also observed in one subject (Figure 13).

**Figure 14:**
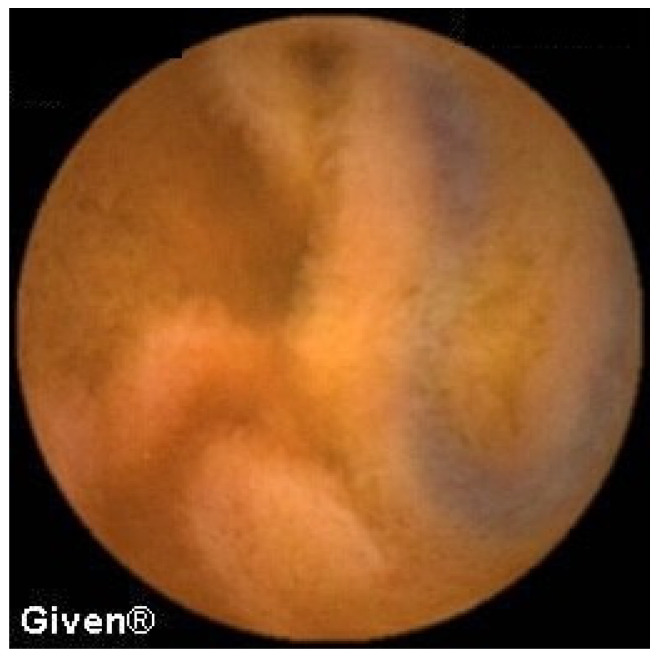
Solitar venectasia.

**Figure 15:**
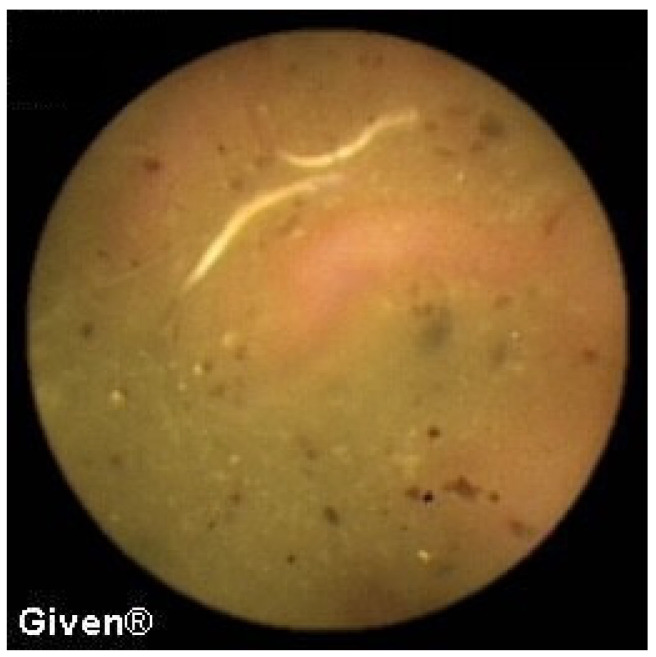
Intestinal parasitosis—Enterobius vermicularis.

In the group with P1 lesions there was one patient with unusual findings that included solitar venectasia (Figure 14) and parasitosis—Enterobius vermicularis, as seen Figure 15.

All patients with P0 lesions were followed up clinically for at least one year; none of them experienced a worsening of symptoms, and no other disease was diagnosed. In the majority of patients (50/67), the bleeding stopped spontaneously, while 17 patients were substituted with oral iron preparations and did not require further blood transfusions.

A detailed list of a suspected SB pathology in patients with other indications for VCE is shown in Table 4, and a diagnosed SB pathology in these patients can be seen in Table 5. In a smaller group of patients examined with VCE due to other indications, 18/47 (38.3%) had P2 lesions, six (12.7%) patients had P1 lesions, and in 23 (48.8%) patients VCE findings showed P0 lesions.

## 4. Discussion

VCE is a disposable, wireless, miniature camera that allows for a direct visualization of the gastrointestinal mucosa. It is a painless, noninvasive procedure, well-tolerated by patients. Originally M2A (“mouth to anus”—from mouth to anus; Given Imaging Ltd., Yoqneam, Israel), VCE was approved in 2001 by the FDA as an “aid” for the visualization of the small bowel mucosa. Then, in 2003 VCE was approved as a “first line” diagnostic tool for the detection of abnormalities of the SB [8,10]. The M2A capsule was renamed to PillCam SB (SB—“small bowel”), following the development of the esophageal video capsule (PillCam ESO) also by Given Imaging. Nowadays, several VCE are available (PillCam SB3, PillCam for Crohn’s system (Medtronic Ltd., Dublin, Ireland), EndoCapsule (Olympus Corp, Tokyo, Japan), MiRoCam capsule (Intromedics, Seoul, Republic of Korea), and the CapsoCam (CapsoVision, Inc., Saratoga, CA, USA) [10,11,12]. In our study, VCE was conducted using the M2A capsule, the Pill Cam SB, and the Pill Cam SB2.

### 4.1. Indications for VCE

OGIB is the main indication for VCE [13] since it attributed to 5% of all GI bleedings. OGIB is defined as persistent or recurring GI bleeding without obvious etiology after negative initial esophagogastroduodenoscopy, colonoscopy, and/or radiological evaluation of the SB (such as SBFT or enteroclysis). Based on the presence of clinically evident bleeding, OGIB is divided into occult (no visible blood) and overt (continued passage of visible blood, such as haematemesis, melaena, or haematochezia) bleeding [8]. Based on the localization, OGIB can arise in the upper (proximal to the Treitz ligament), middle (from the Treitz ligament to the terminal ileum), or lower parts (distal from the terminal ileum) of the GI tract. In more than 80% of cases, the cause is thought to lie in the small intestine and can arise from a number of conditions, including vascular lesions, tumors, Meckel’s diverticulum, and inflammatory lesions [8,9,14]. Until recently, the diagnostic algorithm for these patients was complicated and involved the application of numerous modalities, including invasive ones. VCE, as a sophisticated and elegant technology due to its non-invasiveness and good tolerance, has become the preferred diagnostic procedure in this indication worldwide, and most medical societies recommend it as the first-line method for exploring the source of OGIB in patients without obstructive symptoms [13]. VCE and balloon-assisted enteroscopy (BAE) have enabled the identification of obscure bleeding sources in the GI tract in most cases and have contributed to a major change in the diagnostic and therapeutic endoscopic approach in patients with obsure bleeding [8]. Thus, clinical guidelines from the American College of Gastroenterology (ACG) proposed that the term ‘small bowel bleeding’ should replace the previously used classification of OGIB. The term obscure is limited to patients without an identified source of bleeding after a thorough examination of the entire gastrointestinal tract, including the small bowel [15]. So far, numerous studies have shown its remarkable effectiveness in detecting the cause of bleeding originating from the small intestine [7,10,13], especially when it is applied at the moment of active bleeding. In our study the most common indication for VCE was OGIB (occult and overt) in 78% of patients.

### 4.2. Diagnostic Yield of VCE

The overall yield of VCE for OGIB has been reported to be in the range of 30 to 70% [16,17,18,19,20,21,22,23,24,25,26,27,28,29,30,31,32,33,34,35,36]. The overall diagnostic yield of VCE in our study was 66.2% that is in agreement with previously published studies. A large meta-analysis by Liao Z. et al. included 227 studies with 22,840 procedures, 66% of which were performed due to suspected small bowel bleeding. The detection rate for VCE in patients with suspected small bowel bleeding was 61% [13]. In our study the diagnostic yield, when P1 and P2 lesions are considered positive findings, for obscure/occult bleeding and iron deficiency anemia was 60.4%, which is in agreement with previously published data from Contaldo et al. [18]. In another study of 911 patients with suspected small bowel bleeding, 56% of patients had a positive definite finding on VCE, the most common one being small bowel angiodysplasias [28].

VCE has a higher accuracy of identifying small bowel pathology than barium small bowel radiography [19,26] and “push enteroscopy” [16,17,21,22,23,24]. In a randomized trial from Laine et al., 136 patients with OGIB were assigned to either VCE (*n* = 66) or small bowel barium radiography (*n* = 70). The diagnostic yield was higher for VCE compared to barium radiography (30% vs. 7%, respectively) [26]. Triester et al. included 14 observational studies in their meta-analysis that compared VCE with other tests for suspected small bowel bleeding. They estimated that the overall yield of VCE was significantly higher than that of push enteroscopy and barium studies (63% vs. 26% vs. 8%, respectively) [30] In the clinical comparative study by Ella and Remke, conventional procedures (X-ray of the small intestine, blood pool scintigraphy, Meckel scintigraphy, angiography, and “push enteroscopy”) identified only five pathological findings in 32 patients (16%), 9 findings (21%) by “push enteroscopy” and 21 findings (66%) by VCE [21]. Lewis et al. indicated that the VCE was superior to push enteroscopy, X-ray small bowel passage, and colonoscopy with retrograde ileoscopy [16]. In this analysis, VCE found a pathological finding in an average of 70% of the 530 pooled examinations. In all publications VCE has been shown to be far superior to all other diagnostic modalities [16,17,18,19,20,21,22,23,24,25,26,27,28,29,30,31,32,33,34,35,36,37,38,39]. In our study, “push enteroscopy” was performed before the capsule in 30 patients, but no positive findings were found in any patient. Enteroclysis was previously performed in 15 patients (5 X-ray and 10 CT enteroclysis), and an underlying pathology was identified in three patients—in two patients Meckel’s diverticulum and in one patient Crohn’s disease was suspected (a thickening of part of the ileal wall and lymphoid agregates were seen). All suspected finding were confirmed by VCE.

Angiography is the most successful procedure when patients have actively bleeding lesions and requires at least 0.5–1 mL/min of blood loss. Tc 99m Er-labeled scintigraphy is usually used as a first diagnostic tool since it has better sensitivity than angiography (it detects bleeding lesions with 0.1–0.4 mL of blood/min). In a study by Saperas et al., angiography was shown to have a lower detection rate of arterio-venous malformations (AVMs) compared to VCE, DBE, and intraoperative enteroscopy and an approximately high miss rate for other lesions in the small bowel that were not actively bleeding [40]. In our study, angiography was performed in six patients, and scintigraphy with technetium pertechnetate in seven patients, but the findings were negative in both procedures (including two patients in whom Meckel’s diverticulum was subsequently detected by VCE).

CT enterography has a good diagnostic yield in the evaluation of patients with small bowel diseases. However, it cannot detect subtle small bowel mucosal lesions [15]. A meta-analysis of the CT enterography results for OGIB that included 18 studies and 660 patients revealed a diagnostic yield of 40% for OGIB patients, which is in accordance with another study that confirmed the lower diagnostic yield of CT enterography compared to VCE (34% vs. 53%, respectively) [41].

In OGIB the diagnostic yield of DBE was similar to VCE [42,43]. The meta-analysis by Teshima et al. included 10 published studies involving 651 patients with OGIB, and it showed no statistically significant differences in the diagnostic yield between VCE and DBE (62% for VCE vs. 56% for DBE), while the diagnostic yield of DBE was found to be better when DBE is performed after VCE [42]. Thus, VCE can serve as a preliminary diagnostic tool in patients with small bowel bleeding, and DBE should be considered in a highly selected group. In our center, DBE has been performed since 2007, and 17 patients underwent DBE before VCE. In eight patients, the finding on the VCE was positive, while the DBE finding was negative (even in four patients in whom tumors were observed by VCE). In five patients, the findings of VCE and DBE were similar. In four patients, the finding of VCE was negative, while that of DBE was positive.

### 4.3. When to Perform VCE-Timing of the Procedure?

The diagnostic yield of VCE is highest when it is performed as soon as possible after bleeding is identified [28,31,35,37,38,44,45,46]. This was postulated almost 10 years ago in the study by Penazzio [31]. Patients were categorized into three groups; the diagnostic yield of VCE was highest in the group with ongoing overt bleeding (92%t) compared to those with previous overt bleeding (13%) and occult bleeding (44%). The most common findings were angiodysplasia (29%) and Crohn’s disease (6%). The authors concluded that the best candidates appear to be patients with ongoing overt bleeding or occult bleeding.

The European Society of Gastrointestinal Endoscopy (ESGE) guidelines also recommend VCE to be performed as soon as possible after a bleeding episode (within 14 days) in patients with OGIB [39]. Our results correlate with this conclusion of the above-mentioned studies. There were significantly more positive findings among OGIB patients with overt GI bleeding than among those with occult GI bleeding (48.8% vs. 30.2%). Other factors associated with an increased yield of VCE include male sex, older age, current hospitalization, transfusion requirements, and the presence of connective tissue disease [28,46].

### 4.4. Findings on VCE

#### 4.4.1. Vascular Lesions

Angiodysplasias are the most common findings in patients with OGIB, with a rate of 50%, followed by inflammatory ulcers (26.8%), neoplastic lesions (8.8%), and the presence of fresh blood (7.7%) [13]. In our study, the overall yield of VCE for OGIB (i.e., yield for any small bowel findings) was 66.2%, and the most common P2 lesions were vascular anomalies, tumors, Crohn’s disease, and Meckel’s diverticulum, which is in agreement with previously reported data [13,16,22,23,24].

#### 4.4.2. Inflammatory Bowel Disease—Crohn’s Disease

VCE can be useful in diagnosing Crohn’s disease in patients with suggestive symptoms and in patients with known Crohn’s disease in order to detect active disease or to evaluate therapy response. The overall diagnostic yield of VCE in patients with known or suspected Crohn’s disease was 55% in a meta-analysis by Liao [13]. VCE should not be used in patients with known or suspected strictures, and pre-procedure evaluation is recommended [47,48,49,50,51]. A SBFT does not necessarily exclude strictures. Capsule retention has been described in up to 13% of patients who underwent a capsule study for known Crohn’s disease, even after performing an initial small bowel study [48]. VCE retention occurred more often in patients with known Crohn’s disease compared to those with suspected Crohn’s disease (5–13% vs. 1–2%) [48,52]. Thus, it is recommended that patients with known small bowel Crohn’s disease who are at high risk of having strictures have small bowel imaging or a patency capsule study prior to VCE. The patency capsule should also be considered in patients with a history of abdominal/pelvic radiation, frequent NSAID use, and previous small intestinal surgery. In patients who are at lower risk, who have a history of small bowel Crohn’s disease, and who are asymptomatic, evaluation with computed tomographic (CT) or magnetic resonance imaging (MRI) enterography is an acceptable alternative to a patency capsule. Many studies have compared VCE with other modalities for small bowel Crohn’s disease. A meta-analysis by Dionisio et al. found that VCE had an overall yield of 50–70% for findings of Crohn’s disease. The yield was higher when compared to other diagnostic modalities, such as SBFT (22%), ileocolonoscopy (48%), push enteroscopy (8%t), or CT enterography/CT enteroclysis (31%) [53]. VCE was also compared with CT/NMR enterography in patients without small bowel strictures [54]. VCE had a sensitivity of 100% for detecting ileal Crohn’s disease, superior to CT enterography (76%) and with a trend toward higher sensitivity than MR enterography (81%). The diagnostic yield of VCE for Crohn’s disease in any portion of the small bowel did not differ significantly from the other studies, but it did detect more cases of Crohn’s disease proximal to the ileum. The study by Flamant showed that the presence of jejunal lesions detected by VCE was associated with an increased risk of a relapse [55]. We diagnosed small intestine Crohn’s disease in three patients, in whom presence of only a few small, flat, but typical aphtous ulcers could not have been diagnosed with other diagnostic modalities. Clinical remission was achieved in all patients after adequate medical treatment. An emerging role of VCE is in the assessment of mucosal healing in Crohn’s disease [56].

The interpretation of findings in patients with suspected Crohn’s disease is complicated. The unresolved and debatable questions include the following: Do all the patients with erosions and/or ulcerations have Crohn’s disease? Are these findings due to NSAID use or are they just part of the “normal” spectrum without clinical significance, as described in some studies in more than 14% of patients [57,58,59]? Part of the answer was provided by Maiden et al., who enrolled healthy volunteers in their study and demonstrated that no erosions were observed in the small intestine after 4 weeks of NSAID abstinence [59]. The impact of the VCE on the further treatment plan of these patients will also depend on the accuracy of the description of the lesions found by the VCE. The interpretation and comparison of previous reports on the diagnostic yield of VCE was limited due to the lack of a standardized and valid scoring system for describing inflammatory lesions in the small intestine (in terms of their size and severity) [60]. In order to overcome this challenge, a scoring system was offered to evaluate the mucosal inflammatory disease detected by the video capsule. This score index is included in the RAPID^®^ 5 Access software [61].

#### 4.4.3. Neoplastic Lesions

Primary neoplasms of the small intestine represent a rare group of tumors that account for 3–6% of all and 1–3% of malignant gastrointestinal tract tumors [30]. The diagnosis of the tumor is made relatively late, on average more than 10 months from the onset of the first symptoms. After the development of VCE, the incidence of small bowel tumors increased by 2–10% [62,63]. In patients with OGIB examined by VCE, tumors are the third most common finding after vascular and inflammatory pathology [13]. Barkin et al. presented large series of patients with histologically confirmed capsule-detected small bowel tumors [64]. Malignant tumors represented 61% (54/89) of those, and benign 39% (35/89). They were most often localized in the jejunum, with the most common malignant tumors being adenocarcinomas, carcinoids, metastatic melanomas, lymphomas, and sarcomas, and the most common benign tumors being GIST-omas, hemangiomas, hamartomas, and adenomas. The largest percentage of patients was investigated for OGIB, but tumors were also diagnosed in patients with chronic abdominal pain, suggesting that VCE should also be considered in these patients. We diagnosed 22 tumors, seven GIST, three neuroendocrine tumors, three metastases of malignant melanoma, two Non-Hodgkin’s lymphoma (NHL), two adenocarcinomas of the proximal jejunum, and one leiomyosarcoma of the medial ileum. Four patients did not accept suggested surgical exploration or further follow-up. Adenocarcinomas are the most frequent malignant tumors of the small intestine, and we diagnosed only two in the proximal jejunum. This can partially be explained by their localization—50% of them are localized in the duodenum, which can also be detected by conventional endoscopy [65]. VCE has the potential to be useful in the early diagnosis of small bowel tumors, thereby providing a greater chance of cure. It also affects the further treatment plan because most patients undergo surgical resection, whether for malignant or benign tumors. VCE cannot evaluate the transmural nor the extraluminal status, so the diagnostic rate might be lower than that of CT or MR imaging [66]. In this situation, MR had a higher specificity than VCE, and CT enterography had a higher sensitivity than VCE [67,68]. In a small study that included 17 patients with small bowel tumors who underwent both CT enterography and VCE, CT enterography was more sensitive than VCE to detecting small bowel tumors (94% vs. 35%) [69]. Lesions in the duodenum and proximal jejunum are easily missed because of the rapid transit of the capsule through these areas. Sometimes transient bulges in the small bowel lumen may appear to be submucosal masses [70,71,72,73]. The main disadvantage of VCE is that it does not permit tissue sampling. It should not be performed in patients in whom small bowel obstruction is suspected since the capsule may become lodged proximally to the obstruction and may require enteroscopy or laparotomy for retrieval, as has been the case in 10–25% of patients with small bowel tumors in reported series [74,75].

#### 4.4.4. Celiac Disease

Celiac disease is a very interesting indication for VCE, but most of the studies included a small number of patients, and larger studies are needed [76,77,78,79,80,81,82,83]. Findings on VCE that are considered consistent for the diagnosis of celiac disease are villous atrophy, fissures, mosaic-like mucosa, flat mucosa due to the absence of visible villi, abnormal villi—thickened and shortened, stacking of folds, and nodularity. VCE enables the assessment of the distribution of disease in the small intestine [76,77]. In the meta-analysis, the sensitivity and the specificity of VCE in celiac disease was 89% vs. 95%, respectively [39]. Generally, VCE is not recommended for suspected celiac disease [82]. VCE should be performed in those patients who do not respond to the gluten free diet in order to exclude complications of the disease. Refractory celiac disease type I showed a smaller diagnostic yield in imaging procedures, including VCE, but extensive mucosal damage seen on VCE could predict the type of refractory celiac disease (refractory celiac disease type II) [80,83]. We had three patients with a near-total atrophy of the mucosa of a proximal jejunum and one patient with a near-total atrophy of part of an ileum, in which the findings on the proximal parts were completely normal both macroscopically and histologically.

#### 4.4.5. Gastrointestinal Polyposis Syndromes

Gastrointestinal polyposis syndromes, such as FAP and Peutz–Jeghers syndrome, are characterized by the presence of multiple polypoid lesions in the GI tract. Neoplasms can occur in more than 75% of patients with Peutz–Jeghers syndrome and in 90% of patients with FAP, and VCE is now widely accepted as a method of screening in these indications [84,85,86,87]. The capsule has a larger diagnostic yield than enteroscopy [86] and detects smaller polyps that are not detectible by radiological examinations [85]. The routine application of VCE in patients with Peutz–Jeghers syndrome is recommended, but its role in patients with FAP remains unclear. Moreover, the capsule can “miss” polyps localized in the area of the papilla due to the presence of bile or the often-rapid transit through the duodenum, and in these patients, duodenoscopy remains the method of choice for follow-up [88,89]. We examined five patients with Peutz–Jeghers syndrome, and small bowel polyps were identified using VCE in four, while in two out of three patients with FAP, VCE detected small bowel polyps.

### 4.5. Use of VCE in Pediatric Patients

Children can also be examined with VCE for the abovementioned indications, and VCE is approved for children older than two years [90,91,92,93,94]. Moreover, only a few limitations in the use of VCE for children are recognized. The main one is the difficulty that swallowing the capsule poses for younger children, which turns VCE into an invasive method as the capsule needs to be delivered into the duodenum using an endoscope under deep sedation or general anesthesia. A special device for the gastroscope was designed in 2004 to assure the direct placement of the capsule into the duodenum in children and adults who do not cooperate or have a disturbed act of swallowing [91,93]. We examined five children, and our youngest patient was 3 years old. In all children, the capsule was placed directly into the duodenum with an endoscope, and all examinations were completed without any complications.

### 4.6. Follow-Up Strategies

A negative finding with VCE is of great importance considering that the negative predictive value (NPV) of the capsule in bleeding is over 90% [31]. This means that patients with a negative result do not have a significant disease of the small intestine, and during follow-up, there is usually no manifestation of new symptoms or worsening of existing ones. During our study period (conducted until 2016), there were no clear-cut off algorithms on how best to follow up patients with OGIB and negative CE. All patients with a negative finding with VCE were followed up for one year, and none of them showed a worsening of their condition or the discovery of a new disease. In our study, bleeding stopped spontaneously in 50/67 patients, while 17 patients were substituted with oral iron preparations and did not require blood transfusions. A meta-analysis by Yung et al. confirmed that a patient with OGIB and negative CE result have a low risk of “re-bleeding“ and that patients can be safely managed with watchful waiting, without further investigation, unless there is a change from occult to overt OGIB or a ≥4 g/dL drop in hemoglobin. Patients who develop re-bleeding after 2 years may need to be invastigated for a new source of blood loss [94]. Another interesting-meta analysis by Tziatzios et al. [95] showed similar re-bleeding rates when comparing Western and Eastern populations, irrespective of either the length of follow-up or the mode (overt vs. occult) of initial presentation. Increased re-bleeding odds after positive (vs. negative) index CE was noticed only in studies originating from the East.

### 4.7. Complications during VCE

Complications during the capsule examination are quite rare and, apart from retention with the development of ileus, do not require surgical intervention [31,96,97,98,99]. Impactions of the capsule at the level of the cricopharyngeal muscle and aspiration into the trachea, which were successfully repaired bronchoscopically, have also been described [98,99]. Most often, however, a retention and prolonged lag of the capsule occurs [98]. A case of spontaneous capsule elimination even 3 weeks after ingestion has been reported in the literature [31]. Therefore, it is not entirely clear what should be done with patients in whom the capsule is retained and no signs of obstruction develop. We had one case of retention with no signs of obstruction. The patient was operated on 7 days after swallowing the capsule, and a benign stenosis was found, one which was not observed in the earlier radiological examination and which was most likely a consequence of taking NSAIDs.

### 4.8. Limitations of the Procedure

VCE, like other diagnostic procedures, has certain disadvantages. The propulsion of the capsule depends on the peristalsis of the intestine, which is variable and sometimes moves the capsule very quickly, so the pathological finding can only be seen in one or several frames. The biggest drawback associated with VCE is the impossibility of taking biopsies, the impossibility of applying therapeutic measures, and “missed” lesions due to the rotation of the capsule and the limited imaging field, and an incomplete examination due to shortened battery life [100].

## 5. Conclusions

VCE is a painless, noninvasive diagnostic procedure which enables the visualization of the entire small bowel mucosa. The procedure is simple, and there is no need for air insufflation and sedation (except in pediatric patients), which is why this method is preferred and comfortable for patients. The most common indication is OGIB, and the best candidates are patients with overt bleeding; patients with IBD should be evaluated in this setting.

## Figures and Tables

**Table 1:** Demographic data.

	Overt Bleeding *N* = 92	Occult Bleeding *N* = 106	Other Indication *N* = 47
Sex (male)	52 (56.5%)	57 (53.7%)	23 (48.9%)
Age (mean, age range)	50 (3–83)	52 (8–83)	40 (17–71)
Past medical history			
Hypertension	18	20	4
Diabetes mellitus	15	8	1
Chronic obstructive pulmonary disease	8	4	/
Chronic renal failure	10	15	2
Cardiomyopathy	5	6	/
Atrial fibrillation	8	10	/
Cerebrovascular insult	/	2	/
Liver cirrhosis	/	/	1
IBD	/	/	2
Drugs			
Warfarin	3	4	/
DOAC	5	6	/
Salicilates	15	18	/
NSAID	5	6	2
Duration of symptoms (in months)	13 (1–72)	21 (2–108)	23 (2–84)
Hemoglobin level prior CE (mean, range g/L)	95 (60–125)	98 (72–151)	110 (85–135)
Previous red cell blood transfusion (Yes)	20	23	/
Number of transfusion prior to CE	2 (1–3)	1 (1–2)	/
Previous hospital admissions due to bleeding (Yes)	24	34	/
Number of previous hospitalizations due to bleeding	1 (1–2)	2 (1–3)	/

**Table 2:** Indications and findings of VCE.

Indication		Number	P2 Lesions	P1 Lesions	P0 Lesions	*p*
Obscure GI bleeding	Overt	92	45 (48.9%)	22 (23.9%)	25 (27.2%)	^a^ *p* = 0.007
	Occult	106	32 (30.2%)	32 (30.2%)	42 (39.6%)	
Other indications		47	18 (38.3%)	6 (12.8%)	23 (48.9%)	

^a^ statistical difference in the frequency of P2 lesions among patients with occult and overt OGIB.

**Table 3:** VCE findings in patient with overt and occult OGIB.

P2 Lesions (*N* = 77)		P1 Lesions (*N* = 54)	
Vascular lesions	31 (40.3%)	Isolated vascular lesions	25 (46.3)
Tumor	22 (28.6%)	Substenosis	7 (13%)
Meckel’s diverticulum	13 (16.9%)	Tumor	6 (11.1%)
Crohn’s disease	3 (3.8%)	Celiac disease	3 (5.6%)
Celiac disease	3 (3.8%)	Crohn’s disease	3 (5.6%)
Erosions	3 (3.8%)	Erosions	3 (5.6%)
Polyp	1 (1.3%)	Meckel’s diverticulum	3 (5.6%)
Colon diverticulosis	1 (1.3%)	Parasitosis	1 (1.8%)
		Lymphoid agregates	1 (1.8%)
		Polyp	1 (1.8%)
		Diverticulum	1 (1.8%)

**Table 4:** Indications and suspected underlying SB pathology excluding OGIB in VCE patients.

Indication for VCE (Clinical Suspicion)	Number	P2 Lesions	P1 Lesions	P0 Lesions
Crohn’s disease	8	2	2	4
Tumors	7	3	0	4
Peutz-Jeghers syndrome	5	4	0	1
Chronic diarrhea	5	2	2	1
FAP	3	2	0	1
Congenital lymphangiectasiae		1	0	2
Polyps	3	0	0	3
Von Hippel–Lindau syndrome	2	0	0	2
Abdominal pain	2	1	0	1
Refractory celiac disease	2	1	0	1
Protein-losing enteropathy	2	0	2	0

**Table 5:** VCE findings in patients with indications other than OGIB for VCE.

P2 Lesions (*N* = 18)		P1 Lesions (*N* = 6)	
Tumor	5 (27.7%)	T cell lymphoma	2 (33.2%)
Peutz–Jeghers sy.	4 (22.2%)	Meckel’s diverticulum	1 (16.7%)
Lymphangiectasie	2 (11.1%)	Celiac disease	1 (16.7%)
FAP	2 (11.1%)	Crohn’s disease	1(16.7%)
Crohn’s disease	2 (11.1%)	Pouchitis	1 (16.7%)
Celiac disease	1(5.6%)		
Portal duodenopathy	1(5.6%)		
Small bowel diverticulosis	1 (5.6%)		

## Data Availability

Dataset available on request from the authors.
